# Perioperative Tranexamic Acid Reduces Bleeding and Wound Complications in Post-Bariatric Abdominoplasty: A Retrospective Cohort Study

**DOI:** 10.3390/life16030519

**Published:** 2026-03-21

**Authors:** Shaghayegh Gorji, Bettina Zidek, Tobias Hirsch, Philipp Wiebringhaus, Maximilian Jacobi, Sascha Wellenbrock

**Affiliations:** 1 Department of Plastic Surgery, University Hospital Muenster, Waldeyer Street 1, 48149 Muenster, Germany; 2 Department of Plastic and Reconstructive Surgery, Institute for Musculoskeletal Medicine, University of Muenster, 48149 Muenster, Germany; 3 Department of Plastic, Reconstructive and Aesthetic Surgery, Hand Surgery, Fachklinik Hornheide, 48157 Muenster, Germany; 4 Department of Plastic, Reconstructive and Aesthetic Surgery—Hand Surgery and Burn Center, University Hospital Schleswig-Holstein, Campus Luebeck, 23562 Luebeck, Germany

**Keywords:** antifibrinolytic therapy, blood loss management, surgical site morbidity, body contouring surgery, patient safety

## Abstract

**Background:** Post-bariatric abdominoplasty is associated with a high risk of bleeding and wound complications due to extensive tissue resection and impaired tissue quality. Tranexamic acid (TXA) reduces perioperative bleeding in multiple surgical disciplines, but evidence in massive-weight-loss abdominoplasty is limited. The aim of our study was to evaluate the association between perioperative TXA use and bleeding-related and surgical outcomes in post-bariatric abdominoplasty. **Methods:** This retrospective cohort study included 97 patients undergoing post-bariatric abdominoplasty, of whom 49 received perioperative TXA and 48 did not. The primary outcome was a composite of bleeding-related complications within 30 days, including hematoma, clinically relevant bleeding, or reoperation. Secondary outcomes included overall and specific surgical site complications, drain output and duration, length of hospital stay, and perioperative hemoglobin changes. Multivariable regression analyses adjusted for body mass index, abdominoplasty type, and year of surgery. **Results:** Bleeding-related complications were significantly lower in the TXA group compared with controls (4.1% vs. 33.3%; unadjusted OR 0.09, 95% CI 0.02–0.40; *p* < 0.001). This association remained significant after adjustment (adjusted OR 0.13, 95% CI 0.03–0.68; *p* = 0.016). TXA use was associated with lower cumulative drain output (median 200 vs. 382.5 mL; *p* < 0.001) and shorter drainage duration (median 4 vs. 5 days; *p* < 0.001). Overall complications were reduced in the TXA group (42.9% vs. 66.7%; *p* = 0.025), driven by fewer wound healing disturbances. Hemoglobin changes, seroma, and infection rates were similar between groups. **Conclusions:** Perioperative TXA use in post-bariatric abdominoplasty is associated with significantly fewer bleeding-related and wound complications without increased adverse effects, supporting its use in this high-risk population.

## 1. Introduction

Surgical procedures in patients with obesity and massive weight loss often require enhanced safety protocols to minimize blood loss and reduce the need for transfusion, thereby improving overall outcomes [1].

The high-risk population following bariatric surgery includes patients who have undergone malabsorptive procedures and face increased risk of nutritional deficiencies (such as vitamin and mineral deficiency, anemia, neuropathy, and osteoporosis); metabolic disturbances (like post-bariatric hypoglycemia, electrolyte abnormalities, secondary hyperparathyroidism, and metabolic bone disease); surgical complications (including anastomotic leaks or strictures, marginal ulcers, and internal hernias); mental health sequelae (such as relapse of depression or anxiety, disordered eating behaviors, and substance use disorders); and obesity-associated medical problems (such as diabetes mellitus type 2, arterial hypertension, and chronic pulmonary disease) [2].

Abdominoplasty is one of the most commonly performed body contouring procedures following bariatric surgery, serving to remove redundant tissue, restore contour, and improve quality of life [3]. According to global statistics of the International Society of Aesthetic Plastic Surgery, abdominoplasty ranked number six for worldwide surgical procedures performed by plastic surgeons with 1,036,236 procedures in 2024 [4]. However, post-bariatric abdominoplasty remains associated with a high risk of complications, including seroma, prolonged drain output, bleeding, hematoma, delayed healing, and skin necrosis, largely due to extensive tissue resection and compromised tissue quality [5]. Although recent studies have investigated the use of tranexamic acid (TXA) in abdominoplasty, reporting reduced seroma formation, aspiration events, and shorter hospital stays [6,7], other studies have failed to show significant benefit, leaving its role in the massive-weight-loss population unclear [8].

TXA, an antifibrinolytic agent that prevents clot breakdown, has been established as an effective therapy to reduce perioperative bleeding in major trauma, postpartum hemorrhage, and a variety of surgical settings [9,10]. Increasing evidence also supports its safety and efficacy in reducing postoperative complications such as bleeding, hematoma, and delayed wound healing across different surgical specialties, including plastic and reconstructive surgery [11,12,13].

Intravenous TXA is safe and may reduce complications, drainage, and hospital stay in abdominoplasty, though more research is needed to define optimal dosing and long-term outcomes; these findings support its potential role in standardized body-contouring protocols [14]. However, evidence regarding TXA use specifically in post-bariatric abdominoplasty is scarce.

Standard abdominoplasty differs from bariatric abdominoplasty as patients who have undergone bariatric surgery constitute a high-risk surgical group. This is attributed to various factors, including significant tissue removal, changes in vascularization, nutritional deficiencies, and reduced capacity for wound healing [15]. Furthermore, the complication rates reported are considerable and frequently surpass those observed in purely esthetic patient groups [16].

Given the growing number of patients undergoing bariatric surgery worldwide and the increasing demand for post-bariatric body contouring, optimizing perioperative safety in this high-risk group is of particular relevance to the field of obesity and related diseases. The present study therefore aimed to evaluate the impact of TXA use in post-bariatric abdominoplasty with respect to postoperative outcomes, complication rates, and length of hospital stay.

As the demand for secondary reconstructive surgeries after bariatric procedures increases, improving perioperative safety for post-bariatric patients is a vital yet insufficiently explored aspect of obesity-related healthcare. Consequently, this study aimed to evaluate the effects of perioperative TXA on postoperative results, complication frequencies, and duration of hospital admission in individuals undergoing post-bariatric abdominoplasty.

## 2. Methods

### 2.1. Study Design

**Figure 1:**
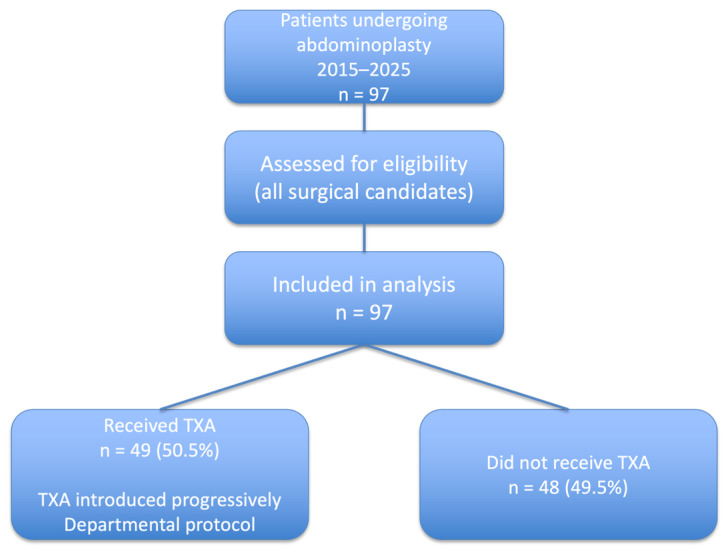
Study flow diagram. A total of 97 patients undergoing abdominoplasty between 2015 and 2025 were included in the analysis. Tranexamic acid (TXA) was progressively introduced during the study period. Following protocol implementation, patients were considered eligible for TXA unless standard contraindications were present. Forty-nine patients received TXA and forty-eight did not.

The primary exposure was perioperative TXA use. Ninety-seven patients underwent abdominoplasty; of these, 49 (50.5%) received TXA and 48 (49.5%) did not (Figure 1). A total of 1000 mg of TXA was administered intravenously at the beginning of each surgery. TXA was introduced progressively in our department. Following implementation of the protocol, all patients undergoing abdominoplasty were considered eligible for TXA unless standard contraindications were present, including a history of thromboembolic events, active coagulopathy, or allergy to TXA. TXA use was therefore guided by departmental protocol rather than strict patient-specific criteria.

In accordance with current guidelines for venous thromboembolism (VTE) prophylaxis, all patients received low-molecular-weight heparin (LMWH) during the perioperative period. The timing and dosing of LMWH administration followed institutional protocols, with the first dose typically given postoperatively once hemostasis was confirmed. Adjustments were made based on individual patient risk factors, including body mass index, associated medical problems, and mobility status. This standardized approach aimed to minimize the risk of VTE while balancing the potential for bleeding complications.

The study period spanned approximately ten years, from January 2015 to October 2025.

Continuous data are reported as mean ± SD or median (IQR), and categorical data as *n* (%). TXA and non-TXA groups were compared using the *t*-test or Wilcoxon rank-sum test for continuous variables and χ^2^ or Fisher’s exact test for categorical variables, as appropriate.

In this study, the extent of missing data for key variables was minimal. All primary and secondary outcome measures were complete for the included patients. Any missing values in secondary variables were documented and accounted for in the analysis. No imputation methods were required, and all available data were used in the statistical evaluation.

The primary outcome was a composite of bleeding-related complications within 30 days (postoperative hematoma, clinically relevant postoperative bleeding, and/or acute reoperation for hematoma/bleeding; yes/no). Secondary outcomes were any surgical site complication, specific complications (seroma, infection, wound healing disturbance, fat necrosis), major complications (Clavien–Dindo ≥ III), cumulative drain output, drainage duration, length of stay (LOS), and perioperative hemoglobin (Hb) change. Absolute Hb drop (g/dL) was defined as pre- minus postoperative Hb; relative Hb drop (%) was recalculated for all patients as (〖”Hb”〗_”pre” − 〖”Hb”〗_”post”)/〖”Hb”〗_”pre” × 100. The association between TXA and the primary bleeding composite was evaluated using multivariable logistic regression, with TXA as the main independent variable. Given the limited number of bleeding events (18/97), the primary model was kept parsimonious and adjusted a priori for key confounders: BMI, type of abdominoplasty (horizontal, inverted-T, panniculectomy), use of antiplatelet/anticoagulant therapy, and year of surgery. Results are presented as odds ratios (ORs) with 95% confidence intervals (CIs). Because of small cell counts, Firth-penalized logistic regression was used as the main model, with standard logistic regression explored in sensitivity analyses.

Binary secondary outcomes were analyzed with similarly structured multivariable logistic regression models, restricted to the same or simpler covariate sets when event numbers were low. Continuous secondary outcomes (drain output, drainage duration, LOS, absolute and relative Hb drop) were analyzed using multivariable linear regression with TXA as the exposure and the same covariates. Distributions and residuals were checked visually; log-transformed models were examined in sensitivity analyses for markedly skewed variables.

### 2.2. Statistical Analyses

All eligible patients in the study period were included; no formal a priori sample size calculation was performed, and results should be interpreted as exploratory. To diminish overfitting in logistic models, the number of covariates was limited in accordance with the available number of events per variable. Analyses were performed on complete cases; missing data patterns were inspected, and sensitivity analyses with multiple imputation may be considered if missingness is substantial for key covariates. All tests were two-sided; *p* < 0.05 was considered statistically significant for the primary outcome. Secondary and exploratory analyses were not adjusted for multiple testing and are reported descriptively. Statistical analyses were conducted using SPSS 30 (IBM Corp., Armonk, NY, USA) and R (R Foundation for Statistical Computing, Vienna, Austria).

### 2.3. Ethics

This study was conducted in accordance with the ethical principles of the World Medical Association Declaration of Helsinki (latest version). The data were retrospectively obtained from institutional databases, anonymized, and de-identified prior to analysis. Given the retrospective design and the use of fully anonymized data, additional approval from the institutional ethics committee was not required.

## 3. Results

### 3.1. Patient Demographics

During the study period, 97 patients were included in the analysis. Of these, 49 (50.5%) received TXA and 48 (49.5%) did not. The mean age of the cohort was 42.2 ± 13.7 years and did not differ between groups (no TXA 42.8 ± 14.1 vs. TXA 41.6 ± 13.5 years). The majority of patients were female (79.4%), with a similar sex distribution in the TXA and non-TXA groups (both 79–80%). Mean BMI at the time of surgery was 27.7 ± 4.3 kg/m^2^ (no TXA 28.1 ± 4.9 vs. TXA 27.3 ± 3.8 kg/m^2^; Table 1).

Most procedures were performed after massive weight loss: 44 patients (45.4%) underwent abdominoplasty following bariatric surgery and 45 (46.4%) after non-bariatric weight loss, with only 8 patients (8.2%) operated on for post-pregnancy or other indications. Median total weight loss was 60 kg (IQR 46–80 kg). Patients in the TXA group had greater preoperative weight loss than those without TXA (median 70 vs. 55 kg), while maximal pre-bariatric weight was numerically higher but comparable between groups (median 150 vs. 139 kg; Table 1).

Associated medical problem profiles were similar between groups. Diabetes mellitus was present in 12 patients (12.4%), hypertension in 15 (15.5%), and 16 patients (16.5%) were current smokers, with no relevant differences by TXA use. Most patients were classified as ASA II; a small number in the TXA group were ASA III, reflecting slightly higher anesthetic risk in this subgroup. The majority of procedures were inverted-T abdominoplasties (58.8%), followed by horizontal abdominoplasties (35.1%) and panniculectomies (6.2%). There was a tendency towards more horizontal abdominoplasties and fewer panniculectomies in the TXA group, but median resection weight was comparable between groups (no TXA 2168 g vs. TXA 2010 g). Only three patients (3.1%) received antiplatelet or anticoagulant therapy preoperatively. Overall, the TXA and non-TXA groups were well balanced across demographics, associated medical problems, and operative characteristics (Table 1).

### 3.2. Primary Outcome: Surgical Complications

Overall, 18 of 97 patients (18.6%) experienced a bleeding-related complication within 30 days. Bleeding events were substantially more frequent in patients who did not receive TXA compared with those who did: 16 of 48 patients (33.3%) in the non-TXA group versus 2 of 49 patients (4.1%) in the TXA group (Table 2). This corresponded to an unadjusted odds ratio (OR) of 0.09 (95% CI 0.02–0.40, *p* < 0.001), representing an absolute risk reduction of 29.3 percentage points and a number needed to treat of approximately four patients to prevent one bleeding-related complication.

In multivariable logistic regression adjusted for body mass index, abdominoplasty type, and year of surgery, TXA use remained independently associated with a lower risk of bleeding-related complications. TXA administration was associated with an 87% reduction in the odds of bleeding events (adjusted OR 0.13, 95% CI 0.03–0.68, *p* = 0.016). In a sensitivity analysis using Firth-penalized logistic regression, the association remained directionally similar but was attenuated and no longer statistically significant (OR 0.41, 95% CI 0.10–1.72). Postoperative hematomas and acute reoperations for hematoma/bleeding were both numerically less frequent in the TXA group, with no acute reoperations occurring among TXA-treated patients (Table 2).

### 3.3. Secondary Outcomes: Drainage, Hospital Length of Stay, and Hemoglobin

Secondary perioperative outcomes are summarized in Table 3. Patients who received TXA had substantially less postoperative drainage and shorter drainage duration than those without TXA. Median cumulative drain output was 200 mL (IQR 90–320 mL) in the TXA group versus 382.5 mL (IQR 200–522.5 mL) without TXA (*p* < 0.001). Drainage duration was reduced from a median of 5 days (IQR 4–6 days) in the non-TXA group to 4 days (IQR 3–4 days) with TXA (*p* < 0.001). Length of stay was also shorter in TXA-treated patients (median 5 vs. 6 days; *p* = 0.002).

**Figure 2:**
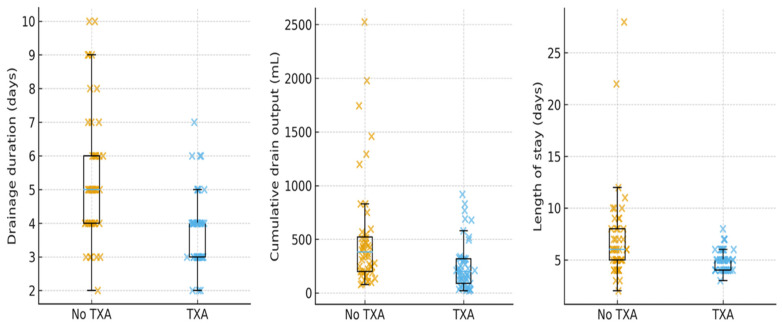
Resource use according to tranexamic acid (TXA) use. Boxplots of (**left**) cumulative drain output, (**center**) drainage duration and (**right**) length of hospital stay according to TXA use, with individual patients shown as jittered points. TXA-treated patients had significantly lower drain output and shorter drainage duration; differences in length of stay were attenuated after adjustment for BMI, abdominoplasty type and year of surgery.

In contrast, TXA use was not associated with relevant differences in perioperative hemoglobin changes. Median absolute Hb drop was 2.2 g/dL in both groups (IQR 1.4–3.1 vs. 1.8–2.8 g/dL; *p* = 0.942), and relative Hb drop remained comparable (15.2% vs. 16.4%; *p* = 0.678). In multivariable linear regression adjusting for BMI, abdominoplasty type and year of surgery, TXA use remained independently associated with fewer drainage days (β −1.22 days, 95% CI −2.02 to −0.43; *p* = 0.003) and lower cumulative drain output (β −220 mL, 95% CI −423 to −16 mL; *p* = 0.035), whereas the reduction in length of stay was attenuated and no longer statistically significant (β −1.00 days, 95% CI −2.64 to 0.64; *p* = 0.23). Hemoglobin changes did not differ significantly between groups in adjusted models (Figure 2).

### 3.4. Complications Other than Bleeding

Overall, 53 of 97 patients (54.6%) experienced at least one postoperative complication. Any complication was more frequent in patients without TXA (32/48, 66.7%) than in those who received TXA (21/49, 42.9%; Table 4). This corresponded to an unadjusted odds ratio (OR) of 0.38 (95% CI 0.16–0.86, *p* = 0.025) for TXA versus no TXA.

The reduction in overall complications was mainly driven by fewer wound healing disturbances in the TXA group. Wound healing problems occurred in 29 of 48 patients (60.4%) without TXA compared with 16 of 49 patients (32.7%) with TXA (OR 0.32, 95% CI 0.14–0.73, *p* = 0.008).

**Figure 3:**
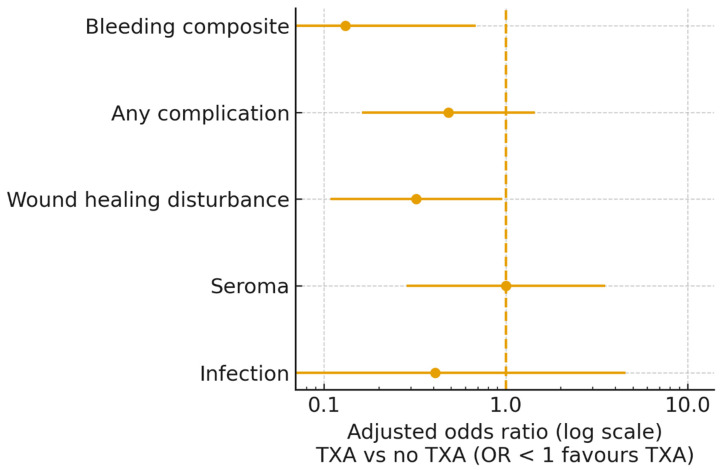
Adjusted association between tranexamic acid (TXA) use and postoperative complications. Forest plot showing adjusted odds ratios (ORs) with 95% confidence intervals for TXA use (vs. no TXA) and key postoperative outcomes. The primary outcome was the composite of bleeding-related complications (postoperative hematoma, clinically relevant bleeding and/or acute reoperation for hematoma/bleeding within 30 days). Secondary outcomes included any complication, wound healing disturbance, seroma and infection. All models were adjusted for body mass index, type of abdominoplasty and year of surgery. The vertical dashed line indicates no effect (OR = 1); ORs < 1 favor TXA.

In contrast, seroma rates were essentially identical between groups (18.8% vs. 18.4%; OR 0.98, 95% CI 0.35–2.71, *p* = 1.00), and infections were infrequent in both groups (6.2% vs. 2.0%; OR 0.31, 95% CI 0.03–3.12, *p* = 0.36). These findings suggest that TXA use was associated with fewer overall and local wound complications, whereas seroma and infection rates were not materially affected (Figure 3).

## 4. Discussion

Our findings demonstrate that perioperative use of TXA in abdominoplasty following massive weight loss is associated with a clinically and statistically significant reduction in bleeding- and wound-related morbidity. Patients receiving TXA experienced substantially fewer bleeding complications, with event rates decreasing from approximately one-third of cases to about 4%, and no acute reoperations for hematoma or postoperative bleeding were observed in the TXA group.

In addition to the primary outcome, TXA use was associated with reduced cumulative drain output, shorter drainage duration, and a shorter hospital length of stay, while perioperative hemoglobin decline remained comparable between groups. Moreover, overall and local wound complication rates were lower among TXA-treated patients, primarily due to a reduction in wound healing disturbances, whereas seroma and infection rates were similar between groups. Collectively, these findings suggest that TXA may favorably influence both perioperative bleeding and local wound outcomes in patients undergoing abdominoplasty after massive weight loss, without evidence of increased seroma formation or infectious complications. The observed reduction in cumulative drain output and drainage duration is of particular relevance in post-bariatric patients, in whom prolonged drainage and seroma formation are common causes of delayed discharge and readmission. This high-risk patient cohort may benefit in particular from improved perioperative safety using TXA.

Our results align with previous studies showing that TXA significantly reduces peri- and postoperative bleeding without increasing cardiovascular risk [17,18,19]. A clinically important benefit of TXA-related blood loss reduction is the decreased need for allogeneic blood transfusions, which leads to significant cost savings primarily attributable to fewer transfusions [20,21]. Wound-related morbidity often arises from the activation of inflammatory pathways, which disrupts normal tissue repair, prolongs healing, and predisposes to chronic wound formation [22]. TXA-mediated reduction in blood loss may alter local fluid dynamics at the injury site, potentially decreasing interstitial fluid accumulation, minimizing edema, and reducing scar formation through inflammation-driven tissue remodeling [23].

Recent evidence from a systematic review and meta-analysis by Monteiro Delgado et al. evaluated perioperative TXA use across 26 randomized controlled trials in general surgery (*n* = 6976) and found that TXA reduced intraoperative blood loss, transfusion requirements, and major bleeding events without increasing thromboembolic risk [24]. Interestingly, in their subgroup analysis restricted to abdominal procedures, these benefits were no longer statistically significant. In the context of post-bariatric abdominoplasty, however, tissue characteristics, extensive dissection, and a high baseline bleeding risk may render TXA more beneficial than in standard abdominal surgery. Our findings support the notion that TXA can meaningfully reduce bleeding-related outcomes in this specific population, highlighting post-bariatric abdominoplasty as a procedure in which TXA may retain clinical relevance even where general abdominal surgery does not.

As an antifibrinolytic agent, TXA is a promising therapeutic agent for reducing perioperative bleeding. Importantly, no thromboembolic events were observed in this study, which is consistent with evidence indicating that TXA does not significantly elevate thromboembolic risk when administered perioperatively in bariatric and other surgical groups. Extensive reviews in bariatric surgery indicate no rise in venous thromboembolism or mortality associated with TXA use, pointing to a generally safe profile in this setting [25]. Careful patient selection is pivotal in post-bariatric populations, given their elevated baseline thrombotic risk from obesity-related associated medical problems and physiological alterations. Furthermore, patients undergoing post-bariatric abdominoplasty may present with nutritional deficiencies related to previous weight loss surgery, which can negatively affect wound healing and increase the risk of postoperative complications. Therefore, thorough preoperative assessment of nutritional status is important in this patient population. Involvement of a hospital nutritionist, particularly in a tertiary care setting, may help identify and correct deficiencies through targeted evaluation and supplementation. Optimization of nutritional status prior to surgery may contribute to reducing wound healing disturbances and other postoperative complications

Some studies report that aside from its inhibitory effect on fibrinolysis, TXA also has an anti-inflammatory effect that may help attenuate systemic inflammatory response syndrome in some patients [26]. These effects include reductions in proinflammatory biomarkers, such as IL-6 and CRP, in surgical patients, as well as attenuation of inflammatory signaling pathways in experimental investigations [27]. Furthermore, several studies suggest that TXA may exert antibacterial effects by inhibiting bacterial aggregation and biofilm formation, which can enhance host immune responses and reduce inflammation, potentially improving the efficacy of antibiotics and supporting wound healing [28]. However, the beneficial effects of TXA are primarily attributed to its antifibrinolytic properties and although anti-inflammatory or antimicrobial effects have been suggested in experimental settings, such mechanisms cannot be inferred from the present clinical data and should be interpreted as hypothesis-generating.

While tranexamic acid is effective in many clinical contexts without increasing thrombotic risk, it may be contraindicated in patients with active intravascular clotting, current thromboembolic disease, or hemostatic imbalances favoring thrombosis [29].

Both intravenous (IV) and topical tranexamic acid (TXA) are supported by clinical data, although IV administration is more widely used and better studied in post-bariatric body contouring surgery. Cohort studies and meta-analyses in abdominoplasty demonstrate that perioperative IV TXA (commonly 1 g, with or without repeat dosing) reduces postoperative drainage, seroma formation, and overall complications without increasing thromboembolic risk [30]. Topical TXA achieves substantially lower systemic absorption and may reduce local bleeding and infection risk; however, evidence for reducing seroma or hematoma is less consistent than for IV use. Accordingly, IV TXA remains the preferred approach in post-bariatric abdominoplasty, with topical TXA as a safe adjunct. While multiple dosing strategies have been described, no studies directly compare single versus repeated IV TXA dosing in post-bariatric abdominoplasty, leaving the optimal regimen undefined.

This study has several limitations. Its retrospective, single-center design and relatively small sample size, with only 18 bleeding events, restrict statistical power and increase the risk for overfitting, despite the use of parsimonious multivariable models and penalized regression in sensitivity analyses. TXA administration was not randomized but introduced progressively into clinical practice over the study period, which may have led to confounding by indication and temporal changes in surgical or perioperative management, even after adjustment for year of surgery and key covariates. Moreover, outcomes and perioperative variables were extracted from routine clinical documentation and may be subject to misclassification or underreporting, particularly for minor complications.

In the context of abdominoplasty, the need for allogeneic blood transfusion is generally very rare when meticulous surgical technique and appropriate postoperative management are applied. In our cohort, transfusion requirements were indeed extremely uncommon. Although tranexamic acid has been associated with reduced transfusion rates and potential cost savings in other surgical fields, this benefit may have limited practical relevance in abdominoplasty. This aspect should be considered when interpreting the potential advantages of TXA in this setting

Wound healing disturbances were defined based on clinical assessment and documented according to the presence of signs such as prolonged wound secretion, wound dehiscence, or local infection requiring medical treatment. As these assessments rely on clinical evaluation and documentation, the reporting of wound healing disturbances may be influenced by surgeon-related factors. In addition, patient-related factors such as associated medical problems and individual variability in wound healing may also affect their occurrence. This should be considered when interpreting the results.

Hematic drainage was assessed based on the overall appearance and volume of the drained fluid; however, the hemoglobin concentration within the drainage was not routinely measured. In addition, no strictly predefined threshold for drainage volume or duration was used to indicate reoperation. The decision for surgical revision was based on clinical judgment, taking into account factors such as persistently high or increasing hematic drainage, the patient’s clinical condition, and suspicion of postoperative bleeding or hematoma formation. This approach may introduce a degree of subjectivity and represents a limitation of the study.

Drainage duration was reduced from a median of 5 to 4 days and length of hospital stay from 6 to 5 days. Although these differences were statistically significant, their clinical relevance should be interpreted with caution. In our department, drain removal was guided by overall clinical assessment and drain output rather than a strict predefined threshold, which introduces a degree of subjectivity. Similarly, the decision for hospital discharge was based on the patient’s clinical recovery and absence of complications. As a result, the observed one-day differences may partly reflect variations in individual surgeon practice and preference. Standardized criteria for drain removal and discharge would help reduce this variability in future studies.

During the study period, approximately 4–5 senior surgeons were performing the procedures in our department at any given time. As the study spans a period of ten years, there were natural changes within the surgical team, which may have influenced surgical outcomes.

Finally, although this study was conducted in a tertiary referral center, the findings may not be fully generalizable to purely esthetic populations and instead reflect the real-world context of post-bariatric reconstructive surgery.

In our sensitivity analysis using Firth-penalized regression, the odds ratio for the primary outcome was 0.41 and was no longer statistically significant, in contrast to the primary model (OR 0.13, *p* = 0.016). This divergence highlights an important uncertainty regarding the robustness of the primary finding and may reflect the relatively low number of events, which can lead to instability in standard logistic regression estimates. While the primary model suggested a significant association, the sensitivity analysis indicates that this effect should be interpreted with caution. These results underscore the need for further studies with larger sample sizes or multi-center designs to confirm the observed association.

Despite clinical use for more than 50 years, investigations into TXA continue to provide updates on applications, efficacy, and risks. In light of ongoing uncertainties regarding optimal dosing and safety, further investigation of this well-established and essential drug is both warranted and timely.

In sum, perioperative TXA use was associated with reduced bleeding, wound-related complications and drainage volume thereby facilitating earlier drain removal and hospital discharge and conserving healthcare resources in post-bariatric abdominoplasty, with no evident safety concerns. Prospective studies are needed to validate these findings and to establish standardized TXA protocols within the post-bariatric reconstructive care pathway.

## Figures and Tables

**Table 1:** Baseline characteristics of patients undergoing abdominoplasty according to tranexamic acid (TXA) use.

Characteristic	No TXA (*n* = 48)	TXA (*n* = 49)	Total (N = 97)	*p*-Value
Age, years, mean ± SD	42.8 ± 14.1	41.6 ± 13.5	42.2 ± 13.7	0.664
Female sex, *n* (%)	38 (79.2%)	39 (79.6%)	77 (79.4%)	1.000
BMI, kg/m^2^, mean ± SD	28.1 ± 4.9	27.3 ± 3.8	27.7 ± 4.3	0.370
Indication, *n* (%)				0.714
Post-bariatric weight loss	19 (39.6%)	25 (51.0%)	44 (45.4%)	
Non-bariatric weight loss	25 (52.1%)	20 (40.8%)	45 (46.4%)	
Other (post-pregnancy/other)	4 (8.3%)	4 (8.2%)	8 (8.2%)	
Weight loss, kg, median (IQR)	55.0 (41.2–68.8)	70.0 (50.0–80.0)	60.0 (46.0–80.0)	0.024
Maximal pre-bariatric weight, kg, median (IQR)	139.0 (132.0–160.8)	150.0 (132.0–169.0)	143.0 (132.0–165.0)	0.203
Diabetes mellitus, *n* (%)	5 (10.4%)	7 (14.3%)	12 (12.4%)	0.759
Hypertension, *n* (%)	9 (18.8%)	6 (12.2%)	15 (15.5%)	0.413
Current smoker, *n* (%)	9 (18.8%)	7 (14.3%)	16 (16.5%)	0.595
ASA class, *n* (I/II/III)	I: 4, II: 37, III: 0	I: 9, II: 29, III: 3	I: 13, II: 66, III: 3	0.053
Type of abdominoplasty, *n* (%)				0.096
Horizontal	13 (27.1%)	21 (42.9%)	34 (35.1%)	
Inverted-T	30 (62.5%)	27 (55.1%)	57 (58.8%)	
Panniculectomy	5 (10.4%)	1 (2.0%)	6 (6.2%)	
Resection weight, g, median (IQR)	2168.0 (1500.0–2772.5)	2010.0 (1350.0–2900.0)	2100.0 (1456.5–2875.0)	0.701
Antiplatelet/anticoagulant therapy, *n* (%)	1 (2.1%)	2 (4.1%)	3 (3.1%)	1.000

**Table 2:** Bleeding-related complications by tranexamic acid (TXA) use.

Outcome	No TXA (*n* = 48)	TXA (*n* = 49)	Total (N = 97)	Unadjusted OR (95% CI) ^1^	*p*-Value ^1^	Adjusted OR ^2^ (95% CI)	*p*-Value ^2^
Any bleeding-related complication ^3^	16 (33.3%)	2 (4.1%)	18 (18.6%)	0.09 (0.02–0.40)	<0.001	0.13 (0.03–0.68)	0.016
Postoperative hematoma	15 (31.3%)	2 (4.1%)	17 (17.5%)	0.09 (0.02–0.44)	<0.001	—	—
Acute reoperation for hematoma/bleeding ^4^	7 (14.6%)	0 (0.0%)	7 (7.2%)	0.06 (0.00–1.01)	0.006	—	—

^1^ Unadjusted odds ratio for TXA vs. no TXA with 95% confidence interval and two-sided Fisher’s exact *p*-value. ^2^ Adjusted for BMI, type of abdominoplasty (horizontal, inverted-T, panniculectomy), and year of surgery in multivariable logistic regression; adjusted ORs are shown only for the primary composite outcome. ^3^ Composite of postoperative hematoma, clinically relevant postoperative bleeding, and/or acute reoperation for hematoma/bleeding within 30 days. ^4^ Defined as any acute reoperation coded for hematoma or postoperative bleeding (including hematoma evacuation) within 30 days.

**Table 3:** Secondary perioperative outcomes according to tranexamic acid (TXA) use.

Outcome	No TXA (*n* = 48)	TXA (*n* = 49)	*p*-Value *
Cumulative drain output, mL	382.5 (200.0–522.5)	200.0 (90.0–320.0)	<0.001
Drainage duration, days	5.0 (4.0–6.0)	4.0 (3.0–4.0)	<0.001
Length of stay, days	6.0 (5.0–8.0)	5.0 (4.0–5.0)	0.002
Absolute Hb drop, g/dL	2.2 (1.4–3.1)	2.2 (1.8–2.8)	0.942
Relative Hb drop, % ^1^	15.2 (9.7–22.9)	16.4 (13.1–21.0)	0.678

Values are median (interquartile range). * Mann–Whitney U test (two-sided). ^1^ Recalculated as $({\mathrm{Hb}}_{\mathrm{pre}}-{\mathrm{Hb}}_{\mathrm{post}})/{\mathrm{Hb}}_{\mathrm{pre}}\times 100$ for all patients.

**Table 4:** Postoperative complications other than bleeding according to tranexamic acid (TXA) use.

Outcome	No TXA (*n* = 48)	TXA (*n* = 49)	Total (N = 97)	Unadjusted OR (95% CI) ^1^	*p*-Value ^1^
Any complication ^2^	32 (66.7%)	21 (42.9%)	53 (54.6%)	0.38 (0.16–0.86)	0.025
Wound healing disturbance ^3^	29 (60.4%)	16 (32.7%)	45 (46.4%)	0.32 (0.14–0.73)	0.008
Seroma	9 (18.8%)	9 (18.4%)	18 (18.6%)	0.98 (0.35–2.71)	1.00
Infection	3 (6.2%)	1 (2.0%)	4 (4.1%)	0.31 (0.03–3.12)	0.36

^1^ Odds ratio for TXA vs. no TXA with 95% confidence interval and two-sided Fisher’s exact *p*-value. ^2^ Any postoperative complication, including bleeding- and non-bleeding-related events. ^3^ Wound healing disturbance as coded in the clinical record (e.g., delayed healing, dehiscence, superficial necrosis).

## Data Availability

The datasets generated and/or analyzed during the current study are not publicly available but can be obtained from the corresponding author upon reasonable request.
